# The role of nurses for patients with Parkinson’s disease at home: a scoping review

**DOI:** 10.1186/s12912-024-01931-y

**Published:** 2024-05-11

**Authors:** Takako Fujita, Miho Iwaki, Yoko Hatono

**Affiliations:** Department of Health Sciences, Faculty of Medical Sciences, Kyushu University, 3-1-1 Maidashi, Higashi-ku, 812-8582 Fukuoka, Japan

**Keywords:** Parkinson’s disease, Nurse role, PDNS, Home care, Community care

## Abstract

**Background:**

Parkinson’s disease is a neurodegenerative disease, and many patients are cared for at home by nurses. Parkinson’s disease nurse specialists have been certified in several countries. This study aimed to provide an overview of what is known about the role of nurses in the care of patients with Parkinson’s disease at home and to determine the differences between nurses and Parkinson’s disease nurse specialists.

**Methods:**

A scoping review was conducted according to the Preferred Reporting Items for Systematic Reviews and Meta-Analyses Extension for Scoping Reviews guidelines. PubMed, Scopus, Web of Science, and Cumulative Index to Nursing and Allied Health Literature were searched (keywords: Parkinson’s disease AND nurse AND [community OR home]) for studies published in English up to September 2023 describing the nurse’s role in caring for patients with Parkinson’s disease at home. Studies without abstracts were removed, along with protocols, systematic reviews, and studies concerned with other diseases or including data that were difficult to distinguish from those of other diseases. Roles were described and organized by category.

**Results:**

A total of 26 studies were included. The nurses’ roles were categorized as overall assessment and support, treatment management, safety assessment regarding falls, care for non-motor symptoms, palliative care, support for caregivers, education for care home staff, multidisciplinary collaboration, and provision of information on social resources. Medication management and education of care home staff were identified as roles of nurse specialists.

**Conclusions:**

This study revealed the role of nurses caring for patients with Parkinson’s disease at home. Because of the complexity of the patients’ medication regimens, nurse specialists provide assistance, especially with medication management and the provision of education to care staff. This study will facilitate the preparation of nurses to acquire the knowledge and skills necessary to help patients with Parkinson’s disease, even in countries where Parkinson’s disease nurse specialists are not officially certified, and will help patients feel comfortable with the care they receive.

## Background

Parkinson’s disease (PD) is a neurodegenerative disease affecting 8.5 million patients worldwide as of 2019 [1]. PD is more common in older people, and the risk increases over time [2–4]. There are prevalence differences by country even within the same ethnic group [4].

Patients with PD experience both motor and non-motor symptoms. Oral medication, levodopa–carbidopa intestinal gel (LCIG), apomorphine management, deep brain stimulation (DBS), and non-pharmacologic therapy, such as rehabilitation, can relieve motor symptoms. Oral medication is used as the first-line treatment based on previous evidence [5, 6].

Nurses play an important role in supporting patients with PD, and nurses specializing in PD or related diseases (PD nurses) have been certified in several countries. The United Kingdom was the first nation to implement this system, and a competency framework that outlines the competencies for each level of nurse has also been developed [7]. In the United Kingdom, PD nurse specialists (PDNSs) run clinics in hospitals or the community, and they visit patients’ homes if the patients cannot attend clinics [7]. However, some countries have not officially approved such certification, even though the number of patients and treatment complexity has been increasing. Therefore, those countries may need to introduce certification in the future.

Many patients are cared for at home by nurses, and the basic treatment is oral medication. As stated previously, some countries have not introduced qualifications for PD nurses. Additionally, nurses in hospitals and clinics only provide care within their departments, and home-visiting nurses only provide care to patients in their own homes or in care homes. Some countries have guidelines for nurses specializing in PD, but these are not focused only on home-based care

This review aimed to provide an overview of what is known about the role of nurses in caring for patients with PD at home, and to determine the differences between nurses and PD nurses caring for these patients.

## Methods

### Design

A scoping review was conducted according to the Preferred Reporting Items for Systematic Reviews and Meta-Analyses Extension for Scoping Reviews guidelines [8]. Because the focus of this study was on the role of nurses in caring for patients with PD at home, a scoping review was conducted. We employed the PCC framework to identify the review question [9], as follows:

- P (Population): patients with Parkinson’s disease
- C (Concept): the role of nurses
- C (Context): the home environment

All types of studies about the role of nurses caring for patients with PD at home were considered. The review protocol was not registered, in accordance with the guidelines.

### Search methods

The PubMed, Scopus, Web of Science, and Cumulative Index to Nursing and Allied Health Literature databases were searched with the keywords Parkinson’s disease AND nurse AND (community OR home). All types of studies published up to September 2023 were included, as the qualifications of PD nurses vary among countries and the role of nurses needs to be understood in broad terms.

### Inclusion and/or exclusion criteria

**Fig. 1:**
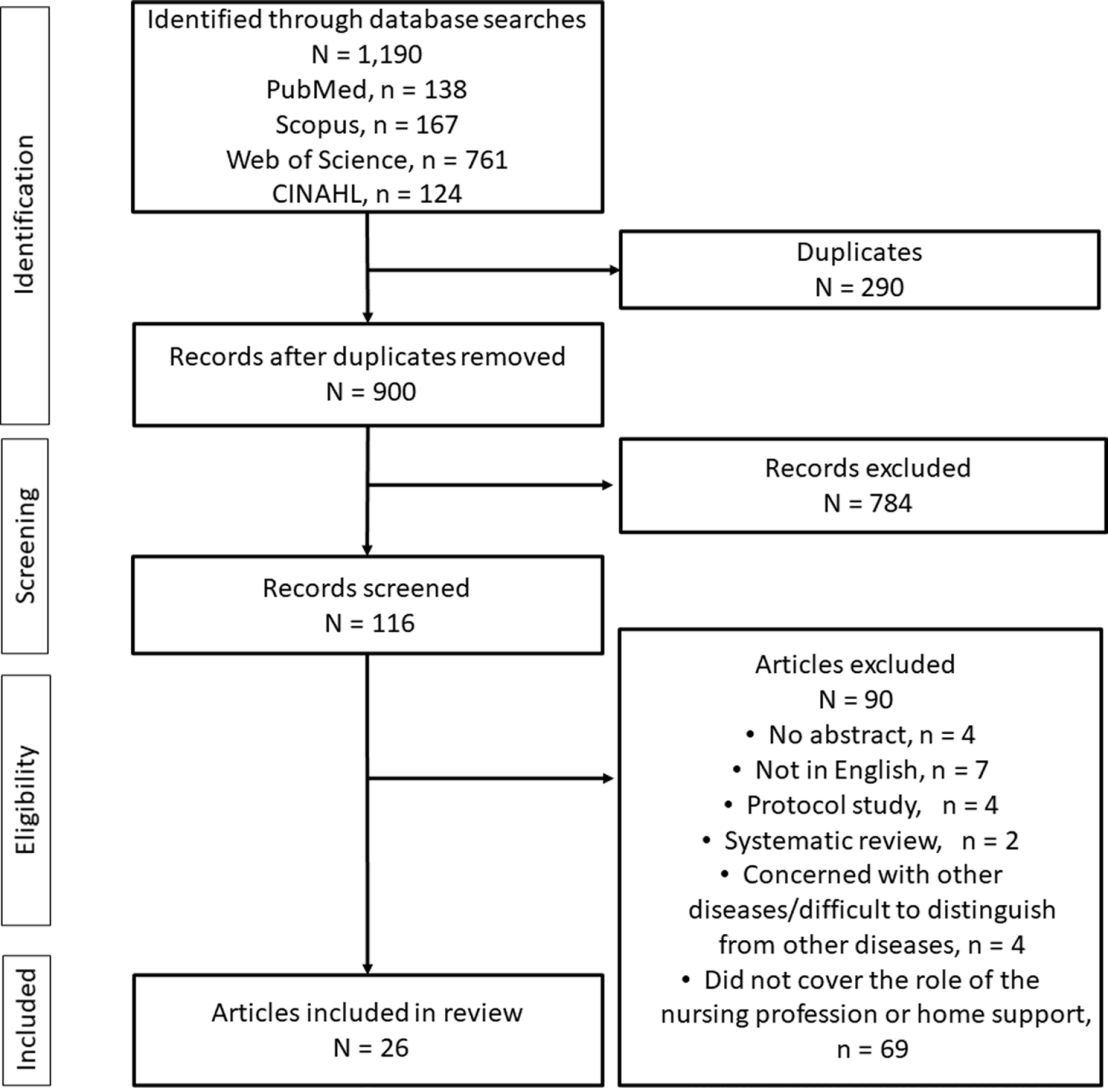
Flow diagram of the study

All references of interest were imported into EndNote 21 (Clarivate Analytics, Philadelphia, PA, USA). Of the 1,190 references extracted during the search, 900 remained after removing duplicates (Fig. 1). Those without abstracts and those written in languages other than English were then removed, along with protocols, systematic reviews, studies concerned with other diseases or including data that were difficult to distinguish from those of other diseases, and studies that did not discuss the role of the nursing profession or home support. Two researchers determined whether studies should be excluded, and another researcher was added to the team to adjudicate for studies that caused a difference in opinion.

### Search outcome

Only English-language literature describing the nurse’s role in caring for patients with PD at home was considered. Roles were described and organized by category.

### Quality appraisal

Quality appraisal is optional according to the guidelines for a scoping review; therefore, quality assessment of each study was not performed [8].

### Data abstraction

For each of the selected studies, we listed the author, year of publication, country in which the study was conducted, objective, study design, participants and sample size, type of nurse, and roles of the nurses. The roles of nurses were organized and described based on the thematic content.

### Synthesis

All themes relating to the nurses’ roles were extracted from each study by three researchers. The researchers discussed and organized the roles based on similarities. The thematic content of the extracted roles was integrated into the narrative.

## Results

The included studies were published between 1999 and 2023 and performed in the United Kingdom (*n* = 12), United States (*n* = 7), Australia (*n* = 2), Sweden (*n* = 1), Germany (*n* = 1), Italy (*n* = 1), China (*n* = 1), and Japan (*n* = 1). PD nurses are referred to as PDNSs in the United Kingdom, Italy, and Germany; as community Parkinson’s nurse specialists (CPNSs) or community-based nurses specializing in PD in the United Kingdom; as registered PD nurses in China; as Parkinson’s nurse experts or movement disorder nurses in Australia; as neuroscience nurses in the United States; and as Duodopa nurse specialists in Sweden. A summary of the selected studies is presented in Table 1.

**Table 1:** Summary of studies included in this review

Authors (Year)	Country	Aim	Study design/methods	Participants	Nurse type	Content
Brock P, et al. [33]	United Kingdom	To understand the perspectives of three service providers (care home staff, PD specialist nurses, and other staff involved in the care of Parkinson’s patients in the UK) in relation to how they provide support for people with PD in care homes, the authors undertook a survey to solicit their experiences	Survey questions (quantitative, qualitative)	60 care home staff 138 PD specialist nurses 68 staff involved in the care of Parkinson’s patients in the UK	PDNS	Education for care home staff Multidisciplinary approach
Chen Y, et al. [32]	China	To identify the barriers to and factors facilitating the provision of palliative care services to PD patients based on a social-ecological theoretical framework to improve the quality of the services	Semi-structured interviews	5 PD clinicians, 7 registered PD nurses, 8 patients, 5 caregivers, and 4 policymakers	Registered PD nurse	Palliative care Multidisciplinary approach Provision of information on social resources
Chenoweth L, et al. [34]	Australia	To determine the impact of a Parkinson’s medicine education program on nurses’ knowledge and practices in two settings where people with PD are cared for: hospitals and residential aged care facilities	Pre-/post-test follow-up design	127 residential aged care facility nurses	Parkinson’s nurse expert	Education for care home staff
Coady V, et al. [25]	Australia	To elucidate rural people with PDs’ preferences for specialist care and explore their experiences with a newly introduced movement disorder nurse program.	Semi-structured interviews	21 people living with PD	Movement disorder nurse	Medication adjustment Support for mental health Support for caregivers Provision of information on social resources
Connor KI, et al. [11]	United States	To examine the quality and frequency of activities during implementation of the CHAPS protocol in a real-world setting	Descriptive study for one intervention group	140 community-dwelling veterans with PD	Nurse care manager	Comprehensive assessment and support Medication adherence Support for mental health
Cotterell P [12]	United Kingdom	To explore the causes and clinical progression of idiopathic PD using an evidence-based approach	Descriptive	Not applicable	PDNS	Comprehensive assessment and support Medication adherence Safety management for motor symptoms Multidisciplinary approach Provision of information on social resources
Drey N, et al. [21],	United Kingdom	To explore how people with PD adhere to prescribed medication and the antecedents of non-adherence to antiparkinsonian medication	Exploratory qualitative study using semi-structured interviews	15 consecutive patients not yet in the advanced stages of PD living at home	PDNS	Medication adjustment
Duffley G, et al. [28]	United States	To evaluate the ability of home health care for postoperative management of DBS patients to reduce travel burden and improve access	Open-label randomized clinical trial	19 randomized patients with PD scheduled for DB receiving the standard of care and 23 randomized patients with PD scheduled for DB receiving home healthcare	Home health nurse	Postoperative management of DBS
Duncan D [30]	United Kingdom	To assess the role of the community nurse in caring for patients with PD and lower urinary disorders	Descriptive	Not applicable	Community nurse	Support for excretory problems
Eggers C, et al. [35]	Germany	To analyze whether a community-based, open-label, integrated approach improves quality of life in PD patients	Randomized control trial	132 patients with PD receiving an individually tailored therapy plan and additional home visits and 125 patients with PD receiving standard German neurological treatment	PDNS	Multidisciplinary approach
Fincher L, et al. [14]	United States	To determine the usefulness and usability of telehealth (telephone and videophone) medication counseling	Mixed-methods study	49 patients with PD	Neuroscience nurses	Medication adherence
Fischer PP [15]	United States	To assess the role of the community health nurse in caring for PD in the US healthcare system	Descriptive	Not applicable	Community health nurse	Medication adherence Safety management for motor symptoms Support for excretory problems Provision of information on social resources
Fleisher J, et al. [18]	United States	To describe a novel, interdisciplinary home visit program specifically designed for individuals with PD and related disorders, and their family caregivers, built upon best-practice principles in the care of multimorbid older adults	Retrospective chart review	85 individuals with PD and related disorders	Registered nurse	Medication adherence Safety management for motor symptoms Support for caregivers Multidisciplinary approach
Fleisher JE, et al. [16]	United States	To describe retention in and satisfaction with the program over 1 year; disease progression of advanced, homebound individuals over time; and whether the program can stabilize quality of life despite expected functional decline	Prospective cohort study	27 patients with PD	Nurse	Medication adherence Safety management for motor symptoms Multidisciplinary approach
Gardner R [13]	United Kingdom	To describe the activities of a PDNS in community care	Descriptive	Not applicable	PDNS	Comprehensive assessment and support Multidisciplinary approach
Gregory P, Morgan K, Lynall A [29]	United Kingdom	To consider the practicality and benefits to patients of transferring evidence-based sleep management skills to community health professionals	Semi-structured interviews	19 PDNSs, 19 occupational therapists	PDNS	Support for sleeping disorders
Iwasa Y, Saito I, Suzuki M [31]	Japan	To analyze nursing records and nursing practice observation records and determine the kind of care currently being provided to patients with PD in their own homes and nursing homes	Questionnaire	21 patients with PD at or above Hoehn and Yahr stage III	Visiting nurse	Support for excretory problems Support for caregivers
Jarman B, et al. [24]	United Kingdom	To determine the effects of community-based nurses specializing in PD on health outcomes and healthcare costs	Randomized controlled trial	1,859 patients with PD	Community-based nurses specializing in PD	Medication adjustment Safety management for motor symptoms Support for caregivers Multidisciplinary approach Provision of information on social resources
MacMahon DG [22]	United Kingdom	To discuss the importance of community care to PDNSs	Descriptive	Not applicable	PDNS	Medication adjustment Support for excretory problems Support for mental health Palliative care Support for caregivers Multidisciplinary approach Provision of information on social resources
Mancini F, et al. [23]	Italy	To explore the hypothesis that adding telenursing to usual care could fill a gap in currently available services, including offering patients easy accessibility to a nurse with specific expertise in PD	Intervention study	1 patient with PD	PDNS	Medication adjustment Safety management for motor symptoms Support for dysphagia Support for mental health
Oyler SE, et al. [19]	United States	To examine the number and types of medication errors detected by a registered nurse during interdisciplinary home visits for patients with advanced PD	Intervention study	26 patients with PD	RN	Medication adherence
Patel K [17]	United Kingdom	To optimize medication for PD with dysphagia	Descriptive	Not applicable	Community nurse	Medication adherence Support for dysphagia
Soper C [20]	United Kingdom	To discuss the interventions provided to a patient over a 6-month period, by a CPNS working alongside a community multidisciplinary team, to improve her medicine concordance and ensure her safety	Intervention study	1 patient with PD	CPNS	Medication adherence Medication adjustment Support for mental health Education for care home staff Multidisciplinary approach Provision of information on social resources
Thomas S, MacMahon D [10]	United Kingdom	To explore common problems associated with PD and implications for nurses working with patients who display symptoms	Descriptive	Not applicable	Nurse	Comprehensive assessment and support Support for excretory problems Multidisciplinary approach
Todd A, James CA [27]	United Kingdom	To draw on both published research and experience from clinical practice to provide a useful resource for community nurses who are involved in the care of patients on apomorphine infusion therapy	Descriptive	Not applicable	Community nurse PDNS	Apomorphine management
Willows, T. et al. [26]	Sweden	To show the feasibility of telemedicine for LCIG home titration, evaluate the use of resources, and assess patient, neurologist, and nurse satisfaction	Observational study	15 patients with PD who are motivated and confident to test LCIG home titration	Duodopa nurse specialist	LCIG infusion care

CHAPS, Care Coordination for Health Promotion and Activities in Parkinson’s Disease; CPNS, community Parkinson’s nurse specialist; DBS, deep brain stimulation; LCIG, levodopa–carbidopa intestinal gel; PD, Parkinson’s disease; PDNS, Parkinson’s disease nurse specialist

In the following section, we describe the identified roles of nurses caring for patients with PD at home.

### Comprehensive assessment and care

Comprehensive assessment is required of nurses not only for patients with PD but also for general patient support. Nurses need to plan appropriate care for patients with PD covering communication, personal hygiene, mobility, constipation, swallowing and diet, and psychological problems [10]. In addition, nurses provide education to the patients, including on therapeutic medications and fall prevention [11].

PDNSs are responsible for supporting patients by conducting regular in-person assessments, considering possible complications, and identifying changes in motor and non-motor symptoms to prevent worsening of the disease [12, 13].

Because worsening idiopathic PD is likely to affect some aspects of a patient’s health and cause serious problems that can lead to hospitalization, such as dysphagia resulting in a chest infection or fractures following falls, an accurate assessment of symptoms by the nurse and an understanding of the concerns of patients with PD and their families and caregivers can help ensure timely responses to emerging problems [12]. In addition, since patients with PD are knowledgeable about their symptoms and the impact of idiopathic PD, PDNSs can consider patients’ concerns and involve them in decision-making at every stage [12].

### Therapeutic management of motor symptoms

#### Support for medication adherence

Medication management is one of the most common forms of support provided by nurses to patients with PD [11]. The support given by both PD nurses and other nurses includes discussing medication management, providing information and education about PD medications and side effects, and reviewing medication schedules [11, 14–17]. In a study conducted in the United States, nurses performed a detailed medication reconciliation, reviewing multiple medication management methods (e.g., pillboxes, timers) and prescriptions, over-the-counter medications, and supplements, and comparing the actual regimen to that listed at the most recent outpatient visit [16, 18]. Patients can also be supported by PD nurses in these respects through telenursing. Neuroscience nurses in the United States provided videophone and telephone guidance, which is helpful in confirming medications, side effects, and schedules [14]. The nurse–patient relationship was reportedly strengthened by this strong individualized support, and patients appreciated the guidance, especially the visual aspect provided by videophone [14].

Because oral medications for patients with PD are complex, it is necessary to be aware of a possible decline in medication adherence. A US medication error study involving registered nurses who made home visits to patients with PD reported that the most common medication errors were taking medications that were not known to their care providers, taking medications that patients were instructed to discontinue, taking an incorrect number of doses, and omitting or forgetting to take a dose [19]. Medication delays can have serious consequences, including impaired physical function and dysphagia, and neuroleptic malignant syndrome can be life-threatening. PDNSs need to be constantly vigilant to ensure that PD medications are not unexpectedly discontinued for any reason [12]. CPNSs may also consult with pharmacies about medication management and introduce dispensing aids, such as reminders and blister packaging [20]. One reason for poor medication adherence may be dysphagia. Crushing tablets or opening capsules to make swallowing easier for patients with PD should not be implemented without seeking advice from the dispenser [17]. Therefore, community nurses should consult with the pharmacist to discuss whether other forms of medications can be used when swallowing difficulties are affecting adherence [17].

#### Medication adjustment

Only studies regarding the adjustment of oral medication by PD nurses were extracted. In these studies, medications were adjusted based on knowledge of the association between diet and medications, including the time between meals and medications, and the timing of oral medications was adjusted to reduce drowsiness as a side effect [14, 21]. PDNSs may also adjust medications. Studies in the United Kingdom and Italy have shown that PDNSs provide information about medical conditions and the purposes of medications, change medications via formal consultations or by telephone, aim to detect problems early, including side effects, when treatment is changed, and advise on medication adjustments [20–23].

A study that conducted semi-structured interviews reported that patients were satisfied with the information provided by PDNSs [21]. A study in which patients were questioned by community-based nurses specializing in PD, which included patients with and without regular interventions who had their clinical health status and response to treatment monitored (reporting back to their general practitioner or consultant as needed), reported that the intervention group had significantly better scores for overall health [24]. In a case study, a CPNS in the United Kingdom observed a patient’s hallucinations and discovered that they were a side effect of L-dopa, which led to an adjustment in dosage [20]. In Italy, a PDNS identified unpleasant hallucinations during telenursing, determined that they were possible side effects of cholinesterase, and provided this information to the physician, which led to modification of the prescription medication [23]. Similarly, in Australia, a movement disorder nurse conducted advocacy regarding medications, and medication was changed after physician–patient interactions [25].

#### LCIG care

LCIG care was reported in one study conducted in Sweden that implemented care at home using telehealth. In that study, Duodopa nurses provided training in the use of the equipment before and during the introduction of telemedicine, support through home visits, and instruction to patients using a video communication system; patients, neurologists, and nurses were all satisfied [26].

#### Apomorphine management

One study in the United Kingdom reported on apomorphine management. A community nursing team was responsible for the daily management of apomorphine, supported by guidance from PDNSs. The role of community nurses in apomorphine management was to supervise and support patients, caregivers, and family members, including by selecting appropriate sites for the infusion, setting up the pump, and siting and removing the needle. Apomorphine users, nurses, and caregivers should follow best practices to minimize severe reactions to apomorphine infusion, and better documentation of the rotation of the infusion site and nodule severity assures a high standard of care [27].

#### Postoperative management of DBS

In a US intervention study, home health nurses supported the postoperative management of patients who had undergone DBS. Postoperatively, trained nurses visited the patients at home to measure vital signs, implement an app-based program, and administer medications and clinical rating scales. The study reported no significant differences in motor symptoms, PD symptom severity, or quality of life compared with outpatients [28].

### Safety assessment related to falls

As patients with PD are at increased risk of falling because of motor and non-motor symptoms, treatment, and other factors, they need to be assessed in terms of safety. Therefore, it is important to identify motor fluctuations [12]. Specifically, nurses use the Unified Parkinson’s Disease Rating scale (I and II) and fall assessments, paying particular attention to tremors; falls; frozen gait; dysphagia; independence level in dressing, toileting, and walking; and environmental safety assessments at home, such as in the bathroom and kitchen, including assessments of clutter and uneven floors [16, 18]. In addition, nurses check orthostatic vital signs given the prevalence of orthostatic hypotension [16]. Because conditions change over time, PDNSs have a role in providing strategies for adapting to changes and advice for maintaining safety, such as by avoiding falls [12]. The appropriateness of mobility aids such as canes and walkers should also be assessed, and it is essential to educate family members about these aids [15].

In a study from the United Kingdom, it was reported that there were no significant differences in motor symptoms or quality of life between patients treated by community-based nurses specializing in PD and those treated by general physicians, and the nurse group had significantly better overall health scores [24]. An Italian telenursing service operated by PDNSs reported 13 telephone contacts during a 3-month intervention period and, at enrollment, 99 falls in the previous 3 months; this decreased to 3 falls in the 3 months after the intervention [23].

### Support for non-motor symptoms

#### Support for sleep disorders

Sleep disorders are also associated with quality of life and the risk of falls in patients with PD. One study from the United Kingdom found that after PDNSs received education on helping patients with PD and sleep disorders, patients reported a reduction in anxiety over sleep problems, feeling able to manage their sleep, having a sense of control over their sleep, and experiencing improved sleep efficiency (time to fall asleep) and quality of life [29]. This education included recognition of sleep problems in patients with PD, health education on sleep and insomnia, sleep assessment, sleep hygiene practices, use of stimulus control and sleep restriction procedures, implementation of relaxation methods, and cognitive approaches to manage insomnia symptoms [29].

#### Support for Dysphagia

Dysphagia is also associated with the previously mentioned oral medications. One role of community nurses for patients with PD in the United Kingdom is to be aware of the risks of dysphagia, to actively ask patients if they have experienced swallowing difficulties, and to consult with pharmacists about the shape and other aspects of the medication, given that difficulty swallowing can affect medication adherence [17]. In Italy, a case study reported that after 13 telenursing sessions over 3 months, in which a PDNS provided symptom assessment and individualized advice by telephone, the initial moderate dysphagia completely disappeared at the follow-up interview 3 months later [23].

#### Support for excretory problems

Urinary problems and constipation need to be incorporated into the nursing plan because of their impact on patient distress [10, 15]. For urinary problems, a comprehensive assessment should be conducted before an appropriate treatment plan is implemented, including a continence assessment conducted with a trained professional and supporting families with tailored bladder training, such as a regular toileting routine, pelvic floor muscle training, and the supply of products for incontinence [30]. Regular clinical monitoring, medication management, and good communication with patients can help empower them to make decisions and choices related to their care [30]. It was reported that urinary problems were common in patients with Hoehn and Yahr stage V, and urinary and indwelling bladder catheters were commonly required [31].

#### Support for mental health

Mental health support and counseling are provided by nurses and PD nurses [11, 22], and were among the most frequently performed nursing activities for patients with PD in a study of nurse care managers in the United States [11].

In terms of specific individualized support, in the United Kingdom, CPNSs found that patients continued to fall, lost weight because of anorexia, and felt increasingly depressed; the nurses discussed the best way forward with multidisciplinary staff and family members [20]. In Australia, a movement disorder nurse provided emotional support to her patient, with a reduction in illness uncertainty achieved through the sharing of information about future expectations [25]. In a case report about a telenursing service provided by a PDNS in Italy, 13 telephone support calls in 3 months led to a reduction in depression and anxiety [23].

### Palliative care

Palliative care is designed to provide specialized disease management and physical, psychological, spiritual, and social support, to reduce suffering and improve quality of life for patients and their caregivers [32]. PDNSs play a role in symptom reduction and pain relief for both patients and caregivers, for example, by addressing dysuria, constipation, and sedation in patients and providing counseling, education, and advice for both patients and caregivers, including by facilitating referrals and collaborations with other agencies [22].

In China, a study of factors promoting and inhibiting palliative care provision by registered PD nurses was conducted, and the facilitatory factors included the desire for palliative care knowledge among healthcare professionals, the presence of social support, nurses acting as a bridge between the patient and physician, convenient community services, and the availability of hospital–community–family based services [32]. Factors inhibiting palliative care included misconceptions about this form of care among healthcare professionals, patients, and caregivers (e.g., believing that palliative care does not include surgical or conservative treatments and is similar to hospice care); lack of time for communication between patients and healthcare professionals; lack of specialized palliative care nurses; lack of palliative care referral criteria; lack of information on palliative care resources; and lack of maintenance of continuity of care [32].

### Support for caregivers

Counseling and education about PD are provided to both patients and caregivers by PD nurses and other nurses, both in the home and over the phone, when patients are living at home [18, 24, 31]. In a US study, a program of home visits by several professionals, including nurses, to assess the psychosocial needs of patients and caregivers, along with follow-up by telephone to address any remaining needs, indicated that 98.1% of caregivers were satisfied with the program [18]. Another study reported that nurses mediated between physicians and patients or their families, such as by providing information to caregivers when the patient’s medication was changed [25]. When palliative care is provided, as noted above, PDNSs are responsible for providing counseling, education, and advice to both the patients and caregivers, as well as for facilitating referrals and coordination with other agencies [22].

### Education for care home staff

Patients with PD may need to be admitted to a care home if they have difficulty living at home. Staff providing care to patients with PD find hallucinations, falls, and physical difficulties difficult to manage [33]. In the United Kingdom, 62% of PDNSs reported training care home staff, providing training to staff when a patient who had been in the care of a CPNS was admitted to a care home and providing training in the area of responsibility in collaboration with the Education and Training Officer of Parkinson’s UK [20, 33]. Specifically, they provided materials and information related to the care of patients with PD, regular reviews of patients by PD nurses or general practitioners, and regular training for new employees [33]. An Australian study introducing an educational program (including video presentations, a 1-hour lecture, and a refresher program after 4–6 weeks) for staff in residential aged care facilities, developed primarily by Parkinson’s nurse experts and focusing specifically on therapeutic medication knowledge and management, reported that program implementation increased levels of knowledge [34].

### Multidisciplinary approach

According to the included studies, PD nurses and nurses collaborated with neurologists, pharmacists, movement disorder specialists, social workers, psychologists, physiotherapists, occupational therapists, speech therapists, and other therapists specializing in neurology, as well as with nutritionists, care staff and, in the United Kingdom, other staff involved in caring for Parkinson’s patients, with contact maintained with hospital staff during admission and discharge [12, 15, 16, 18, 24, 33, 35]. Collaboration involved joint home visits, the exchange of information through conferences, and referrals to other professionals such as psychologists and therapists [12, 20, 33].

Nurses need to work as a care team to address both aspects of the clinical environment and the care provided in the home for patients with PD [13]. When working within a multidisciplinary team, nurses need to assess the patient’s abilities and plan appropriate care, including in relation to both written and verbal communication; personal hygiene; mobility (with consideration of problems related to moving in bed, transferring, and initiating and maintaining mobility); toileting, with special attention paid to the possibility of constipation; swallowing and eating; and psychological issues [10]. Nurses play a liaison role, working with the primary care team for ongoing assessment and treatment as needed [24, 32]. The role also includes training nursing home staff and various other clinical and general staff, as well as educating the community [20, 22].

The effects of multidisciplinary collaboration on patients have also been reported. A study conducted in Germany reported clear improvements in emotional well-being, stigma, communication, and physical discomfort through collaboration among PDNSs, community neurologists, and movement disorder specialists [35]. A study of palliative care in China stated that the registered PD nurse should serve as a coordinator between the physician and the patient, as the absence of this collaboration results in poor palliative care [32].

### Provision of information on social resources

Nurses have a role in providing patients with PD, and their families and caregivers, with local and national resources and information, such as recommendations for respite and day hospitalization care, assessment of eligibility for social benefits, and special assistance at home [12, 15, 22, 24]. For example, in an Australian study, movement disorder nurses provided extensive practical support through a variety of strategies, incorporating home modifications and housekeeping support to assist patients with daily living [25]. In a study of palliative care in China, a lack of information on social resources led to a lack of service utilization [32]. Finally, a case study conducted in the United Kingdom reported that a patient who had repeated falls, worsening depression, and difficulty living alone was referred to a nursing home [20].

## Discussion

This study was conducted to provide an overview of what is known about the role of nurses in caring for patients with PD at home, and to determine the differences between nurses and PDNSs caring for these patients. Nine main roles were identified: overall assessment and support, treatment management, safety assessment regarding falls, care for non-motor symptoms, palliative care, support for caregivers, education for care home staff, multidisciplinary collaboration, and provision of information on social resources. Most of the roles were similar to those associated with nursing care at home for patients in general [36] and were performed by both nurses and PD nurses. The results suggest that the main role of PD nurses at home is to manage medication and educate care staff. While these are also important roles for PD nurses in clinics, PD nurses in the home environment can provide superior support because they understand patients’ daily lives. The results of this study will help nurses prepare by providing them with the knowledge and skills needed to help patients with PD, leading to more reliable care for patients, especially in countries lacking a PD nurse system.

Because the basic treatment for PD is oral medication, medication management by nurses, subsuming medication adherence and dosage adjustment considering side effects and timing, is considered to be particularly important in supporting home care. Most patients with PD, particularly those with early stage disease, engage in medication non-adherence behaviors, often intentionally [21, 37]. One common reason for hospitalization among patients with PD is falls or fractures caused by motor symptoms; in fact, this accounts for 65% of all falls [38]. For preventing hospitalization, nurses should be aware that patients are likely to show non-adherence behaviors, and they should aim to prevent missed or incorrectly taken medications by using medication boxes, building a relationship in which the patient feels free to ask for help regarding medication problems, and possibly providing guidance to family members and other support persons. The role of medication dosage adjustment was extracted only from the studies about PD nurses included in this review. It would be particularly meaningful for PD nurses to be involved in this aspect of care because they might spend more time with patients than other professionals; they can assess side effects and the need to add, subtract, or change medication doses at particular times, as well as explain the situation to the physician. In addition to oral medication, nurses are responsible for the daily management of apomorphine in homebound patients and are supported by guidance from PD nurses [27]. Novel non-oral medications may emerge in the future as alternatives to apomorphine, LCIG, and non-pharmacological treatments such as DBS. PD nurses may be required to advise nurses and to make suggestions to the patient’s physician regarding changes in administration methods and treatments, including implementing other non-pharmacologic therapies.

When patients with PD have difficulty living at home, they may be considered for admission to a care home, especially if they have advanced PD or comorbidities that significantly increase the possibility of admission, such as hip fracture or dementia [39, 40]. Care needs for admitted patients with PD may not be met if staff knowledge of PD-related problems is insufficient [41]. In addition to the complexity of oral medications for PD, patients take medicines for the treatment of comorbidities as needed, which may make it difficult for care home staff without sufficient knowledge to assist patients in taking their medications. Therefore, education of care staff by PD nurses is necessary to ensure the safety of patients with PD.

Other roles of nurses related to motor and non-motor symptoms in patients with PD at home are generally applicable to patients with other diseases as well. Specific details of the nurse’s role regarding constipation, which is one of the non-motor symptoms of PD, were not extracted from the studies included in this review. However, the frequency of constipation varied among the studies, from 7 to 70%, and constipation is considered a common symptom in patients with PD because of the effects of the both the disease itself and oral medications [42]. In the management of constipation, dietary intervention with probiotics and prebiotics and the use of lubiprostone and macrogol have been suggested to be potentially effective, whereas the evidence for the efficacy of abdominal massage is considered insufficient [43, 44]. A nurse intervention program for constipation in patients with PD has been created in China [45], and its results suggests that support for defecation is one of the forms of care that nurses should provide. Sleep medications are sometimes used to treat sleep problems, which are classified as non-motor symptoms. However, in patients with PD, only the dopamine agonist rotigotine has been shown to be effective; evidence for the efficacy of other drugs is insufficient [44]. Among older patients with PD, 18.6% were prescribed hypnotics, with benzodiazepines being reported to increase the risk of injury significantly, and melatonin receptor agonists reportedly significantly increasing the risk of femoral fractures [46]. Therefore, it is important to understand the treatments for non-motor symptoms and to provide nursing care while considering their side effects. This can be expected to improve the quality of life of patients with PD.

In addition to nursing care to address the symptoms of patients with PD at home, we found that nurses need to play roles in multidisciplinary collaborations and provide information on social resources. There are differences among countries and regions in terms of the granting of qualifications and the tasks performed in accordance therewith. This study found that there are PD nurses in the United Kingdom, Italy, Germany, Sweden, Australia, the United States, and China, and a competency framework for nurses working in PD management has been created in the United Kingdom [7]. In the Netherlands, which was not covered by this study, professional qualifications have been granted, and guidelines have been created [47]. Nurse-led community care is also provided in Singapore, particularly for patients with severe motor impairments and those without a caregiver; patients are visited by a PD nurse and, if necessary, referred to relevant community services [48]. In these countries, PD nurses take the lead in supporting patients, which is essential for patients to live at home. By contrast, in Japan, for example, nurses at hospitals and clinics only provide care within their departments, and home-visiting nurses only provide care to patients at home or in care homes. As such, some countries are not able to provide nursing care across all inpatient, outpatient, and home care settings. Instead, Japan has a national qualification for public health nurses, who are mainly affiliated with government agencies, playing a liaison role by providing information on available social services and communicating with various agencies. However, they do not provide patient support under the orders of a physician and cannot provide medical assistance. The introduction of PD nurses in countries such as Japan will require that they be given PD-specific roles in accordance with the national system. Clarifying the roles of each type of care provider in every country may increase the likelihood that patients with PD can continue to live at home.

This review has some limitations. First, we only included articles written in English; references written in other languages or included in databases from other countries were not extracted. On the basis of the distribution of the included studies by country, selection bias is plausible given that the countries with multiple references were all English-speaking countries. Second, no studies in this review described in detail the differences between the roles of PD nurses and other nurses in countries where PD nurses are qualified, and the relative impact on patients of interventions implemented by the different types of nurses was not evaluated. Interventions implemented by PD nurses may lead to greater improvements in patients’ symptom control and quality of life compared with those implemented by nurses. Therefore, further research focusing on the differences in nursing roles and outcomes is needed.

## Conclusions

This study clarified the nine main roles of nurses caring for patients with PD at home, including overall assessment and support, treatment management, safety assessment regarding falls, care for non-motor symptoms, palliative care, support for caregivers, education for care home staff, multidisciplinary collaboration, and provision of information on social resources. Because PD medications are complex, medication management and education for care staff are particularly important roles for PD nurses. This study will help prepare nurses by providing them the knowledge and skills necessary to assist patients with PD, even in countries that do not have a PD nurse system, thereby leading to care that will improve patients’ sense of security.

## Data Availability

Not applicable.

## Abbreviations

PD: Parkinson’s disease
LCIG: levodopa–carbidopa intestinal gel
DBS: deep brain stimulation
PDNS: Parkinson’s disease nurse specialist
CPNS: community Parkinson’s nurse specialists
